# In the SOAP: the Supplemental Offer and Acceptance Program (SOAP) from the perspective of a Community Hospital Residency

**DOI:** 10.3402/jchimp.v2i2.18712

**Published:** 2012-07-16

**Authors:** Stephanie Detterline, Robert Ferguson

**Affiliations:** Internal Medicine Residency Program, Medstar Union Memorial Hospital; Internal Medicine Residency Program, Medstar Union Memorial

The Supplemental Offer and Acceptance Program (SOAP) was developed by the National Residency Matching Program (NRMP) as a way to modernize and streamline the ‘Scramble’ process for unfilled positions during Match Week of the Main Residency Match. As we all know, competition for residency spots is becoming more fierce, and the number of total unmatched applicants to PGY-1 positions (8,794 in 2010) continues to rise as unfilled PGY-1 positions (1,060 in 2010) continue to fall (1). Historically, the Scramble would begin on Tuesday at noon EST and conclude at noon on Thursday. The majority of spots were allotted within 4 hours, and over 90% were filled with 48 hours in 2009 and 2010 (2). In those hours, there was widespread panic, lots of wheeling and dealing and a huge disorganized influx of faxes, telephone calls, and emails.

As many of us had observed in our own experiences with the Scramble, multiple problems were identified by The Scramble Work Group established by the NRMP and AAMC including lack of organizational stewardship and transparency, no consistent or orderly process for applying to programs, and no rules to govern applicant and program behavior. It was also felt that applicants were forced to make career decisions too quickly, often in minutes (3).

So the SOAP was a long time coming, born of a joint task force established in 2008 with representation from medical school student affairs deans, residency program directors, and recent graduates of US and international medical schools (4). Over the next 2 years the process was extensively discussed and commented upon, and it was finally decided by the NRMP board to institute the SOAP during Match Week, 2012. The SOAP was to solve all of the Scramble's problems. A detailed timeline was created. Programs would find out if they filled on Monday, a day earlier than usual. Then would begin a 48-hour period of receiving SOAP applications and conducting ‘interviews.’ Programs created a final list by Wednesday at 11:30, and the eight offer rounds of 3 hours each would commence over the next 53 hours.

Did we know any of this when it mattered most? Nope. We remember hearing about the impending SOAP at national meetings, in regional conferences and at special meetings set up by our own system's GME office... but didn't pay very much attention. The SOAP really wasn't our concern. We had no recent need to acquire any post-match candidates, the program was going strong, esprit de corps was high, and there was no way we'd be joining those poor losers who had to be the guinea pigs for this new SOAP process. Boy, were we wrong?

Monday, March 13, 2012, dawned like every other Monday for the past few years when we find out that we filled our categorical and prelim spots, take a deep breath, and then await the identities of the chosen ones. Too easy. At around 1, there were frantic cries of ‘this must be a mistake’ and ‘gotta be a computer glitch,’ and within the next few minutes it sunk in that we had to SOAP for five prelims. (We've found that it works quite well as a verb.) We learned that 47 preliminary medicine programs across the country did not fill through the NRMP and that each candidate who went unmatched would be able to apply to 25 programs through the SOAP.

Over the next 48 hours, we received almost 1,900 new applications (2,900 when all was said and done by the end of the week), downloaded (or tried to) 30,000 files, made no less than 15 desperate calls to the NRMP with either process questions or computer ‘issues,’ talked with 20 candidates (before giving up on that tactic), reviewed about 200 files, spent A LOT of time with our GME director, and almost lost our residency coordinator to general freaking out.

It was truly enough files to choke a computer. For a moment, we thought we smelled smoke coming out of the desk top.

By Wednesday morning, we had organized ourselves enough to put together a list of 35 candidates. The list was a mixture of knowns and unknowns, students who had applied to both the categorical and prelim matches, US grads and non-US grads. We understood the process much more than we had a mere 48 hours before, but had yet to grasp the nuances of how to rank students and what to expect in this new system. At noon, our list was uploaded and the top five applicants on our list received offers with 2 hours to RSVP. At the end of this period, three had accepted which left two spots unfilled, and there were 25 of the originally 35 people left on our list. Apparently, seven had either dropped out or taken an offer elsewhere.

The SOAP was not over for us.

We had not formed a strategy yet at this point and had also not realized that the original rank list could be reordered, so we sat tight. At 3:00, round 2 began and we offered to the next two students on the list, neither known to us, neither took the spot. There were 18 people left on our list; seven that deserted. We had come to the realization that we were not very good at this game.

The SOAP was not over for us.

Thursday was going to be our day; the day the SOAP ended. By now we had learned how to adjust our list and recognized the importance of having some kind of rapport with the people you've ranked, especially as the number of spots to fill dwindled. You could have your heart set on a pristine candidate, but if they don't know you or what your program is about and they have other options remaining, chances are they are going to take a pass.

At this point, it is important to note that new applications continued to stream in all day every day this week. Every morning, there were hundreds (thousands?) of more applications. The information overload was extreme, as was the sense of being very out of control of the whole process. Seeing all these hopeful candidates whose files we would never get a chance to review really put the whole process into perspective. So we turned away from the computer screen constantly uploading new data, rounded up what we thought were two strong AND likely candidates, and made our offer during SOAP, round 4, on Thursday at 9:00 in the morning.

One offer was accepted.

The SOAP was not over for us.

We had one spot remaining and the overwhelming pressure to make this offer count; we were hoping it would be our last. The next round started at noon. Something caught our eyes. One newly downloaded candidate (had entered SOAP only that morning) stood out, and we had a telephone chat with the student. All seemed promising and the student was placed at the top of the list at 11:45. At 12:05, this student filled our last spot.

Everyone breathed a collective sigh of relief.

The SOAP was over for us.

So, did we like the SOAP? Well, I guess as much as a program could like the process that means they didn't fill their spots in the match. Overall, we do think it is fairer. The process of making a list allows you time to think about your choices and weigh your decisions. If we would have made more of an effort to understand the process, it would have gone much more smoothly. Other lessons learned:

1. Volumes are daunting. There is no way to download that much information in such a short time.
2. Reaching is dangerous. There is little benefit to offering a position to an applicant you have no relationship with.
3. Time consuming – the SOAP stretches the post-match process from hours to days. Need to clear schedules for the week in case you might be in the SOAP.
4. Tech intensive – need to reserve the services of your IT-friendly person for this period.

We knew we were part of a historic moment. I guess we are better for it. But for us, being forced into the SOAP for the crime of not matching was a cruel and unusual punishment. We hope we don't have it next time.

Elsewhere in this issue are nine manuscripts. An original research study raises questions about risk vs. benefit of CT scanning for pulmonary emboli. A second research study looks for a relationship between resident inexperience and poor glucose control. There are two other papers on diabetes that were first presented at the Medstar Union Memorial Hospital 34 Annual Diabetes Symposium in 2011.

There is a perspective public health piece on possible risk of another anthrax bioterrorism. There are two important case reports: a devastating dementia case and a case of organ damage secondary to excessive Vitamin C intake. The ECG column superbly demonstrates a case of WPW pre-excitation syndrome cured in the EP lab with a ‘delta-wave ectomy’. There are excellent radiology images of a carotid artery dissection causing Horner's Syndrome.

*Stephanie Detterline, MD* Associate Program Director Internal Medicine Residency Program Medstar Union Memorial Hospital *Robert Ferguson, MD* Program Director Internal Medicine Residency Program Medstar Union Memorial Editors
